# ITGA3–MET interaction promotes papillary thyroid cancer progression via ERK and PI3K/AKT pathways

**DOI:** 10.1080/07853890.2025.2483379

**Published:** 2025-03-26

**Authors:** Youmian Lan, Dongchen Liu, Bin Liang, Xuhong Song, Lingzhu Xie, Hanwei Peng, Haipeng Guo, Chaoqun Hong, Xuwu Weng, Xiaolong Wei, Xiaoqi Liao, Rui Liang, Dongyang Huang, Muyuan Liu

**Affiliations:** ^a^ Department of Head and Neck, Cancer Hospital of Shantou University Medical College, Shantou, China; ^b^ Department of Cell Biology and Genetics, Key Laboratory of Molecular Biology in High Cancer Incidence Coastal Chaoshan Area of Guangdong Higher Education Institutes, Shantou University Medical College, Shantou, China; ^c^ Department of Central Laboratory, Cancer Hospital of Shantou University Medical College, Shantou, China; ^d^ Department of Pathology, Cancer Hospital of Shantou University Medical College, Shantou, China

**Keywords:** ERK, papillary thyroid carcinoma, PI3K/AKT, ITGA3, MET

## Abstract

**Background:**

Studies have examined the role of integrin α3 (ITGA3) in papillary thyroid carcinoma (PTC). However, the functional and molecular mechanism by which ITGA3 is involved in the progression of PTC remains poorly understood.

**Methods:**

To investigate the role of ITGA3 in PTC, raw PTC transcriptome data underwent comprehensive bioinformatics analyses, including differential expression, co-expression network, and enrichment analyses. ITGA3 expression was validated *via* immunohistochemistry and western blotting in PTC tissues. Cell functional assays and xenograft models assessed PTC cell behaviour. The potential mechanisms of ITGA3 were elucidated using bioinformatics analyses, western blotting, co-immunoprecipitation, and immunofluorescence. Finally, integration of ITGA3 expression with clinical parameters enabled nomogram construction for precise prediction of cervical lymph node metastasis (CLNM) in PTC.

**Results:**

ITGA3 was upregulated in PTC and associated strongly with CLNM (79.5% *vs.* 53.84%, *p* = 0.016). ITGA3 expression enhanced PTC proliferation and migration *in vitro* and *in vivo via* cooperating with the MET protein tyrosine kinase, followed by phosphorylation of MET at Tyr1234/1235, and activation of ERK and PI3K/AKT signaling pathways. Furthermore, upregulation ITGA3 reduced phosphorylation at FAK-Tyr397 and Src-Tyr416 in PTC cells. Finally, a nomogram combining ITGA3 expression and clinical parameters for predicting CLNM was constructed and validated, achieving a ROC curve AUC of 0.719, suggesting potential application for PTC diagnosis.

**Conclusions:**

ITGA3 promotes PTC cell proliferation and migration by cooperating with MET to activate MET-ERK and MET-PI3K-AKT signalling. ITGA3-MET cooperation may serve as a potential therapeutic target.

## Introduction

Papillary thyroid carcinoma (PTC) ranks first among all thyroid malignancies worldwide [1,2]. Although PTC patients generally show good prognosis, about 23.5% of PTC patients relapse or develop metastasis after surgery, endocrine suppression therapy and radioactive iodine therapy [3,4]. Additionally, more than 50% of PTC patients with loco-regional relapse become radioiodine-refractory, which is associated with poor progression-free survival [5–9].

The role of integrin α3 or α3β1 in PTC carcinogenesis has been investigated. Knockdown experiments in TPC-1 cells demonstrated that ZNF367, a member of the zinc finger protein family, regulates cellular adhesion, invasion and migration through, at least in part, modulation of integrin α3 expression [10]. In TPC-1 cells, miR-524-5p inhibits cell viability, migration and invasion, through targeting integrin α3 [11]. Mautone et al. confirmed a role for α3β1 in cell motility and invasion during PTC tumour progression and demonstrated the existence of an altered integrin expression signature that correlates with histopathology, specific driver gene mutations, and aggressiveness of the disease [12]. However, studies on PTC have yet to dissect the detailed α3β1 signalling pathways.

Integrins are the main cellular adhesion receptors that play multifaceted roles as signalling molecules, mechanotransducers and key components of the cell migration machinery [13]. At least 18 α and 8 β subunits are known in humans, generating 24 heterodimers, many of which recognize fibronectin, laminin, vitronectin, collagen and other extracellular matrix (ECM) components that cluster in subcellular structures known as focal adhesions. The majority of integrin heterodimers contain the β1 subunit, which can form heterodimeric complexes with 12 α subunits [14]. A vast collection of literature exists regarding altered integrin expression in different cancer types, where integrins play roles in supporting oncogenic signalling and cell motility, invasion and metastasis [15–19]. The primary intracellular downstream signalling mediators of integrins involve focal adhesion kinase (FAK), Src-family protein tyrosine kinases, and integrin-linked kinase (ILK) [14]. In the cytoplasmic tail of β1 integrins, key tyrosine residues are located within highly conserved NPXY/NXXY motifs, and act as docking sites allowing the recruitment of signalling molecules, such as FAK, ILK and Src kinase [18,20]. Signalling events mediated by integrin ITGA3/ITGB1 (α3β1) are essential for mammary tumorigenesis. Integrin α3β1 plays a crucial role in FAK signalling pathways and promoting malignant proliferation and invasion in diseases such as breast cancer [21], ovarian cancer [22], head and neck cancer [23,24] and pancreatic cancer [18,25].

Previous studies have also reported the crosstalk between integrins and the MET protein tyrosine kinase [20,26–28]. One conventional mode of crosstalk appears to occur through inside-out signalling, whereby MET acts upstream of the integrin receptor, in a manner independent of hepatocyte growth factor (HGF) or in an HGF-dependent manner [20]. MET-integrin cooperation can also occur through outside-in signalling, whereby integrin activation, triggered by binding to its extracellular ligand in the ECM, is also able to promote MET phosphorylation [20,26]. Furthermore, MET-β1 cooperation can occur on endosomes, enabling an inside-in signalling [20,29]. The exact mechanisms of MET phosphorylation downstream of an integrin, however, are not clear.

In the current study, we provide direct evidence that upregulated ITGA3 reduces FAK phosphorylation, but interacts directly with MET to promote MET phosphorylation in an HGF-independent manner, followed by activation of ERK1/2 and PI3K/AKT signalling, and promotion of PTC cell progression, migration and invasion. We also developed a nomogram integrating expression of ITGA3 and clinical parameters for predicting cervical lymph node metastasis (CLNM) with PTC patients.

## Materials and methods

### Clinical specimens and public data sources

Clinicopathological and RNA sequencing data of 507 papillary thyroid cancer tissues and 58 normal adjacent tissues were downloaded from The Cancer Genome Atlas (TCGA) thyroid cancer dataset (THCA), and three additional independent datasets of PTC were obtained from the Gene Expression Omnibus (GEO) database (GSE60542, GSE33630, GSE35570). Seventy-eight paraffin-embedded PTC tissues and 68 normal adjacent tissues, along with 12 pairs of fresh PTC tissues were obtained from the Cancer Hospital of Shantou University Medical College (Shantou, China) from 2018 to 2022. All participants signed an informed consent form. Ethical approval for this study was granted by the Ethics Committee of the Cancer Hospital of Shantou University Medical College (Approval No. 2021069), and conformed to the provisions of the1964 Declaration of Helsinki and its subsequent amendments.

### Bioinformatics analyses

Differentially-expressed mRNA of PTC patients was analysed using the R software limma package. The adjusted *p*-value was analysed to correct the false positive results in TCGA or GEO datasets. Adjusted *p < 0.05* and Log2 (Fold Change) >1 or Log2 (Fold Change)< −1 were used to define the threshold for the differential expression of mRNAs. The statistical difference between two groups was compared through the T test. Box plots were drawn by the R software ggplot2 package. Gene Ontology (GO) and Kyoto Encyclopedia of Genes and Genomes (KEGG) enrichment analyses were conducted using the R software clusterProfiler package and visualized by https://www.bioinformatics.com.cn (last accessed on 10 July 2023), an online platform for data analysis and visualization. The two-gene correlation was visualized by ggstatsplot package.

### Immunohistochemistry

Slides of paraffin-embedded tissues were incubated with primary anti-ITGA3 antibody (1:100 dilution, Rabbit, PA5-82356, Invitrogen) overnight at 4 °C. Image-Pro Plus 6.0 was used to calculate the integrated optical density (IOD) for pathological scoring. Immunohistochemical staining was calculated using the immunoreactive score (IRS) system reported previously [30], resulting in IRSs ranging from 0 to 12. An IRS ≤ 4 was considered low, 4 ≤ IRS ≤ 8 was considered moderate, and an IRS > 8 was considered high expression.

### Western blotting

Total protein was extracted from fresh tissues and cells using RIPA buffer (Invitrogen, USA) supplemented with protease and phosphatase inhibitor (VL312018, Thermo Fisher Scientific, USA). Proteins were resolved by 10% SDS-polyacrylamide gel electrophoresis (SDS-PAGE) and transferred to PVDF membranes, followed by blocking with 5% non-fat dry milk. The primary antibodies used for western blotting are listed in Table S1.

### Cell culture and transfection

Human PTC cell lines (KTC-1, B-CPAP, TPC-1) and a human thyroid follicular epithelial cell line (Nthy-ori3-1) were purchased from the National Collection of Authenticated Cell Cultures (Shanghai, China). The passage number of all cell lines used for the experiments was no greater than 20. All cell lines were maintained in RPMI 1640 medium (RPMI 1640; Gibco, USA) supplemented with 10% fetal bovine serum (FBS; Gibco, USA) at 37°Cunder 5% CO2. For knockdown of human ITGA3, shRNA sequences targeting ITGA3 were cloned into an LV3(H1/GFP ± puro) lentiviral cloning vector (GenePharma; Shanghai, China). The shRNA sequences are shown in Table S2. Lentivirus was packaged in HEK293T cells, following transfection of lentiviral packaging vectors by using Fugene (Promega, France), and then used to infect KTC-1 and B-CPAP cells. For overexpression of ITGA3, premade lentiviral particles were purchased from Shanghai Jikai Gene Chemical Technology Co., Ltd. (GOSL0314773) and transduced into TPC-1 cells. Transfected cells used for knockdown or overexpression were selected with puromycin (2 µg/mL) for 2 weeks, and the expressions of target proteins were confirmed by western blotting.

### Wound-healing assay

Wound-healing assays were performed by seeding 1 × 10^6^ cells per well into a 6-well culture plate and incubating for 24 h. Scratch wounds were produced using a 200 μl plastic pipette tip, and then cells were cultured in RPMI 1640 medium with 0.5% FBS. Wound margins of the same position were recorded by high-resolution photographs at 0, 18 and 24 h. Cell migration was evaluated by measuring the average distance between the margins of cells, at each time point, using Image-Pro Plus 6.0.

### Colony formation assay

Cellular proliferation of PTC cells was assessed by colony formation assay. For this assay, 300 cells per well were plated in 6-well plates and incubated for 7 days at 37 °C. The cells were fixed with 4% paraformaldehyde for 30 min and stained with 0.1% crystal violet for 30 min. Then, the plates were washed with ddH2O and dried before being photographed.

### Transwell assay

We used a transwell assay to measure cell invasion and migration. For this assay, 3 × 10^4^ cells (for the transwell invasion assay) or 5 × 10^4^ cells (for the transwell migration assay) in 200 μL serum-free RPMI 1640 medium were seeded into the upper chamber of an 8-μm pore transwell (3422, Corning, USA). Then 600 μL RPMI 1640 medium containing 20% FBS was added to the lower compartment of the chamber. Cells were allowed to invade through the Matrigel matrix (3445-010-01, R&D Systems) or migrate for 24 h. Migrated cells were fixed with 4% paraformaldehyde for 30 min, stained with 0.1% crystal violet for 30 min, and counted in five randomly selected fields (× 200) under a light microscope.

### Cell proliferation assay

Cell viability was measured daily with a Cell Counting Kit-8 (CCK8) kit (C6050, NCM Biotech) according to the manufacturer’s instructions. PTC cells were seeded into 96-well plates at a density of 2 × 10^3^ cells per well, and incubated for 5 days at 37 °C. For determination of viability, CCK8 was added to the cells for 1 h at the same time each day, and absorbance was measured at 450 nm.

### Xenograft tumour formation in nude mice

Twelve 6-week-old female nude mice were purchased from Beijing HFK Bioscience CO., LTD and randomly divided into two groups: an ITGA3-knockdown group and an shNC group (6 per group). A total of 5 × 10^6^ B-CPAP cells in 100 µl Matrigel were subcutaneously injected into the back of each nude mouse. Body weight of mice and tumour size were measured and recorded every 3 days. Tumour volume was calculated using the formula of 1/2(length × width^2^). After 34 days, all mice were euthanized, and tumours were removed and collected for western blotting.

Animal experiments were approved by the Ethics and Indications committee of Shantou University Medical College (approval No. SUMC2021-098). The animal study followed the Arrive guidelines 2.0 [31].

### Co-immunoprecipitation

Protein–protein binding properties were confirmed by co-immunoprecipitation (Co-IP). Cells were pretreated with RIPA buffer supplemented with protease and phosphatase inhibitor (VL312018, Thermo Fisher Scientific, USA) for western blotting and Co-IP. The supernatants were pretreated with normal IgG and protein A/G magnetic beads (HY-K0202, MCE, USA) for 2 h. After removing the beads, the supernatants were incubated with primary antibodies or normal rabbit IgG overnight, and then incubated with protein A/G magnetic beads for 6 h at 4 °C on a rotating platform. Next, the beads were washed three times with PBST buffer, and the proteins bound to beads were eluted and denatured by boiling for 10 min in 20 µL SDS-PAGE sample loading buffer. The proteins were separated by 10% SDS-PAGE followed by western blotting or silver staining. All antibodies used in the Co-IP are listed in Table S1.

### Immunofluorescence

About 1 × 10^5^ PTC cells were seeded into a 6-well culture plate for 24 h, then fixed with 4% paraformaldehyde for 30 min. Slides were blocked for 30 min in PBS with 1% bovine serum albumin (BSA). Antibodies against mouse MET (3127, Cell Signaling Technology) and rabbit ITGA3 (PA5-82356, Invitrogen) were applied at 1:100 dilution. Sections were incubated overnight with both Abs at 4 °C. After several washes with PBS, secondary antibody against mouse and rabbit at 1:500 was applied for 1 h at room temperature. Slides were mounted with Antifade Mounting Medium with DAPI (P0131-25ml, Beyotime) and kept at 4 °C. The slides were imaged in the Central Laboratory of Shantou University Medical College using a laser scanning confocal microscope (Zeiss LSM800, Carl Zeiss).

### Establishment and validation of a diagnostic prediction model and nomogram for cervical lymph node metastasis

The correlation between CLNM and expression level of ITGA3 as well as clinicopathological parameters was studied using univariate logistic regression. Parameters with important clinical implications in univariate logistic analysis were included into multivariate logistic regression to establish a diagnostic prediction model for predicting CLNM. The predictive accuracy was estimated internally using the C-index, the area under the ROC curve (AUC), calibration curve, decision curve analysis (DCA) curve and net reclassification index (NRI). External validation was conducted by using the SUMC cohort.

### Statistical analysis

All statistical analyses were performed with R software (version 4.1.0) and GraphPad prism (version 8.0). The T test was used to compare two experimental groups. The χ2 test was used to assess differences in clinical features. Disease-free survival rates were estimated by the Kaplan–Meier method. Two-sided *p*-values were calculated, and a value of *p* < 0.05 was considered to indicate statistical significance.

## Results

### ITGA3 is upregulated in PTC tissue and is related to lymph node metastasis

To identify genes that might be co-expression modules and associated with lymph node metastasis for PTC, we analysed PTC transcriptome raw sequences from the cohort in TCGA (*n* = 377) and our own SUMC cohort (*n* = 78). Clinicopathological futures of the SUMC and TCGA cohorts are shown in Table 1.

**Table 1.** Clinicopathological futures of the SUMC and TCGA cohorts.

Characteristics	TCGA Cohort, n (%)	SUMC Cohort, n (%)	*P*
**Gender**			0.150
** Male**	98(26.0%)	14(17.9%)	
** Female**	279(74.0%)	64(82.1%)	
**Age**			>0.999
** ≤55**	263(69.8%)	55(70.5%)	
** >55**	114(30.2%)	23(29.5%)	
**T Stage**			0.526
** T_1-2_**	229(60.7%)	44(56.4%)	
** T_3-4_**	148(39.3%)	34(43.6%)	
**N Stage**			0.012
** N^-^**	186(49.3%)	26(33.3%)	
** N^+^**	191(50.7%)	52(66.7%)	
**M Stage**			0.188
** M_0_**	371(98.4%)	75(96.2%)	
** M_1_**	6(0.6%)	3(3.8%)	
**Focus Type**			–
** Unifocal**	201(53.3%)	–	
** Multifocal**	176(46.7%)	–	
**Disease-free**			–
** No**	27(7.2%)	–	
** Yes**	350(92.8%)	–	

**Figure 1.**
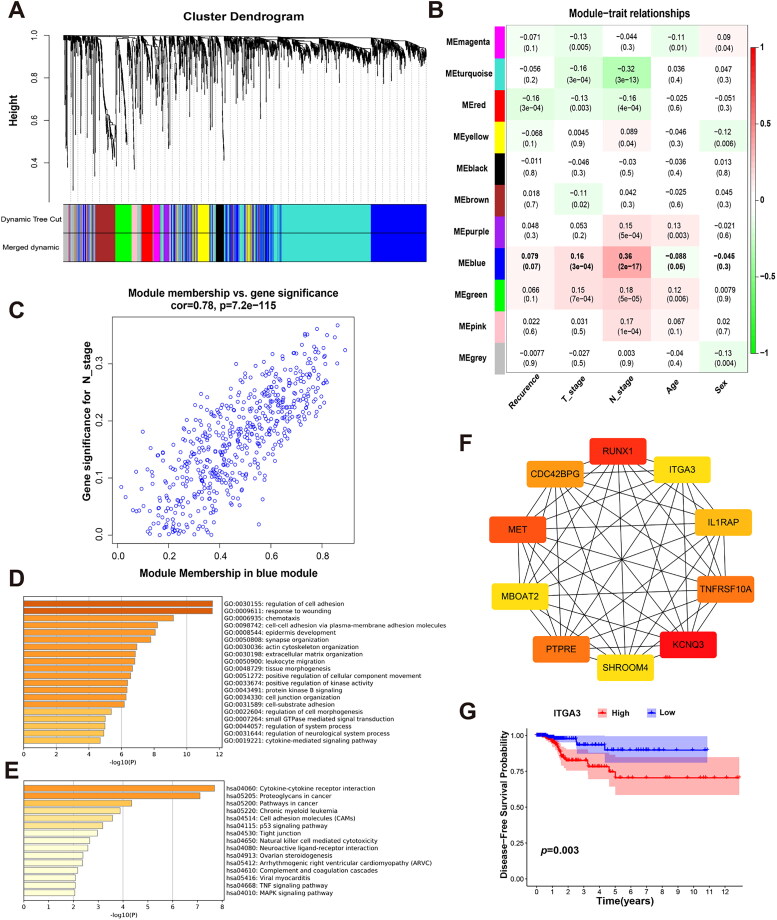
WGCNA analysis and enrichment results. (A) Waterfall plot of WGCNA. (B) Module-trait relationship results. (C) Correlation analysis between N stage and the blue module. (D) GO enrichment of genes within the blue module. (E) KEGG enrichment of genes within the blue module. (F) Ten hub genes from the blue module were identified using the cytoHubba plugin of cystoscope. (G) Disease-free survival curve of THCA patients in TCGA, with a comparison between the low and high ITGA3 expression groups (*n* = 507).

Differential analysis of transcriptome data from PTC tissues and adjacent normal tissues in TCGA was conducted using the edgeR package. Genes exhibiting |logFC| > 1 and *p* < 0.05 were considered statistically significantly differentially expressed. This analysis revealed 5637 differentially expressed genes between the two groups, comprising 3341 upregulated genes and 2296 downregulated genes. Subsequently, all differentially expressed genes were subjected to weighted correlation network analysis (WGCNA), a systematic computational biology approach for analyzing gene expression data [32]. WGCNA involves constructing a gene co-expression network to explore interrelationships among genes, and clustering genes into modules representing sets with similar expression patterns across samples. In our study, a total of 11 co-expression modules were identified (Figure 1(A)). Among them, based on module-trait relationship analysis, the blue module was closely related to N stage, T stage and recurrence (Figure 1(B)). The correlation coefficient between N stage and the blue module was 0.78 with a *p*-value of 7.2 × 10^−115^ (Figure 1(C)). This result implies that genes in the blue module play an important role in lymph node metastasis. Next, the Gene Ontology (GO) and Kyoto Encyclopedia of Genes and Genomes (KEGG) enrichment analyses indicated that genes in the blue module were enriched in pathways involved in cell adhesion, which further suggested that the blue module was strongly correlated to lymph node metastasis. Finally, 10 hub genes (*ITGA3, RUNX1, MET, PTPRE, KCNQ3, CDC42BPG, MBOAT2, SHROOM4, TNFRSF10A* and *IL1RAP)* from the blue module were identified using the cytoHubba plugin of Cytoscape (Figure 1(D–F)). Among these 10 hub genes, 7 (*ITGA3, RUNX1, MET, PTPRE, KCNQ3, MBOAT2, and TNFRSF10A*) were significantly associated with PTC patient disease-free survival (DFS) **(**Figure 1(G) and Figure S1). These results indicate that ITGA3 is one of the hub genes and is associated with DFS of PTC patients.

**Figure 2.**
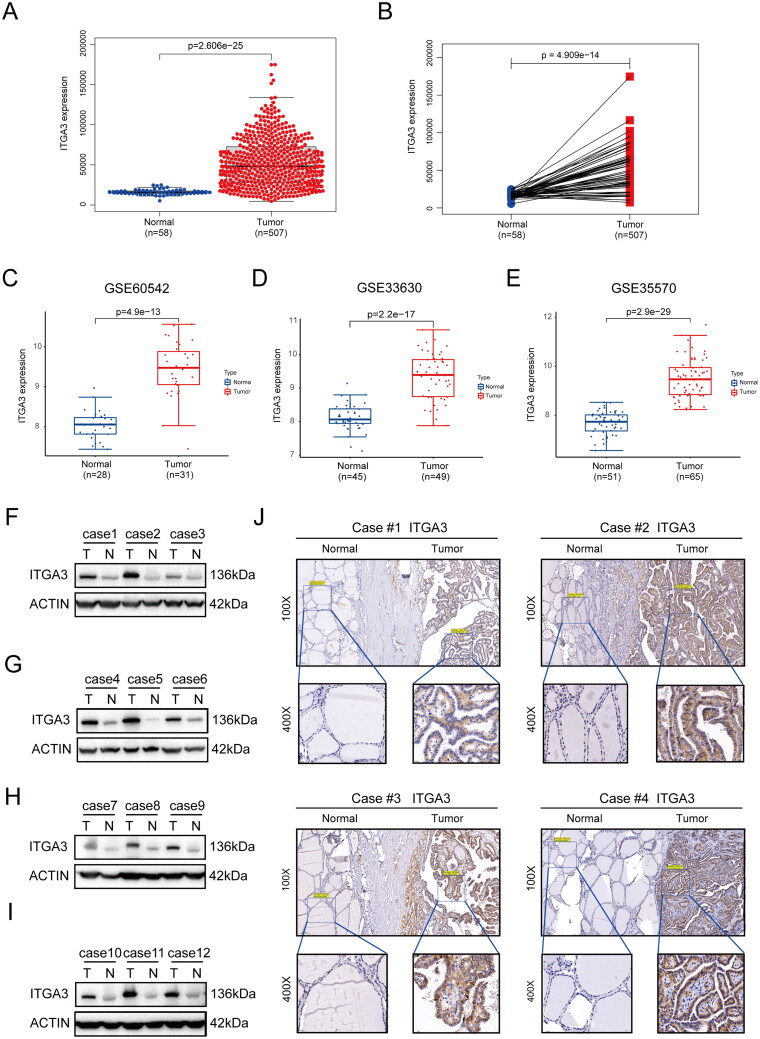
ITGA3 Is highly expressed in PTC. (A) mRNA expression of ITGA3 in the THCA cohort from TCGA (*n* = 507). (B) ITGA3 mRNA expression by paired-difference analysis in the THCA cohort from TCGA (*n* = 507). (C)-(E) ITGA3 mRNA expression in the THCA cohort from the GEO datasets (GSE60542, GSE33630, GSE35570). (F)-(I) Western blotting showing the protein level of ITGA3 in 12 PTC patients from the SUMC cohort. (J) Immunohistochemical staining for ITGA3 in PTC tissues and adjacent non-cancerous tissues in the SUMC cohort (*n* = 78).

Expression of ITGA3 mRNA in PTC tissues was evaluated in the TIMER 2.0 database, and revealed higher expression in multiple tumours, including PTC, than the normal controls (Figure S2). Furthermore, we found that ITGA3 was upregulated in human papillary thyroid cancer tissues (*n* = 507) compared to normal controls (*n* = 58) (Figure 2(A,B)). This result was confirmed by three additional independent PTC datasets from the GEO database (Figure 2(C–E)). Western blotting also revealed significantly higher ITGA3 protein expression in PTC tissues than in case-matched normal tissues (12 out of 12) (Figure 2(F–1)). Immunohistochemistry revealed that ITGA3 was moderately or highly expressed in 56 of 78 (71.8%) PTC tissues compared with 3 of 68 (4.4%) normal adjacent tissues (Figure 2(J), Table 2). Taken together, these results show ITGA3 is upregulated in PTC tissues.

**Table 2.** Immunoreactive scores of PTC tissues and normal adjacent tissues.

Type	N	ITGA3*			*P*
		Low	Moderate	High	
**Normal**	68	65	3	0	<0.001
**Tumor**	78	22	22	34	

*IRS: immune reactive score.

To evaluate the importance of ITGA3 in PTC patients, we analysed the correlation between the expression of ITGA3 and clinical parameters. For TCGA, the results showed ITGA3 was higher in lymph node metastasis-positive patients compared to lymph node-negative patients (*p* = 2.4 × 10^−11^). A similar result was obtained between T_3-4_ stage and T_1-2_ stage patients (*p* = 0.00016). For the SUMC cohort, all patients in our cohort (*n* = 78) were divided into high- and low-ITGA3 groups by the median average optical density (AOD) of ITGA3. Consistent with the cohort in TCGA, the CLNM rate was higher in the ITGA3 high expression group than in the low expression group (79.5% vs. 53.8%, *p* = 0.016) (Table 3). Taken together, ITGA3 is closely associated with CLNM in PTC. Moreover, GSEA shows that 131 gene sets were significantly enriched in the high-ITGA3 group, and most of the genes were strongly correlated with metastasis (Table S3). Taken together, these results show that ITGA3 is closely associated with CLNM in PTC.

**Table 3.** Correlation between ITGA3 and clinical parameters for the SUMC cohort.

Characteristics	N	ITGA3		*P*
		Low (AOD^a^ ≤0.0451)	High(AOD > 0.0451)	
**T Stage**				>0.999
** T_1-2_**	44	22(56.4%)	22(56.4%)	
** T_3-4_**	34	17(43.6%)	17(43.6%)	
**N Stage**				0.016
** N^-^**	26	18(46.2%)	8(20.5%)	
** N^+^**	52	21(53.8%)	31(79.5%)	
**M Stage**				0.077
** M_0_**	75	36(92.3%)	39(100%)	
** M_1_**	3	3(7.7%)	0(0.0%)	
**Sex**				
** Male**	14	3(7.7%)	11(28.2%)	0.018
** Female**	64	36(92.3%)	28(71.8%)	
**Age**				
** ≤55**	55	26(66.7%)	29(74.4%)	0.456
** >55**	23	13(33.3%)	10(25.6%)	

**^a^**AOD: average optical density.

### ITGA3 promotes PTC proliferation and migration in vitro and in vivo

**Figure 3.**
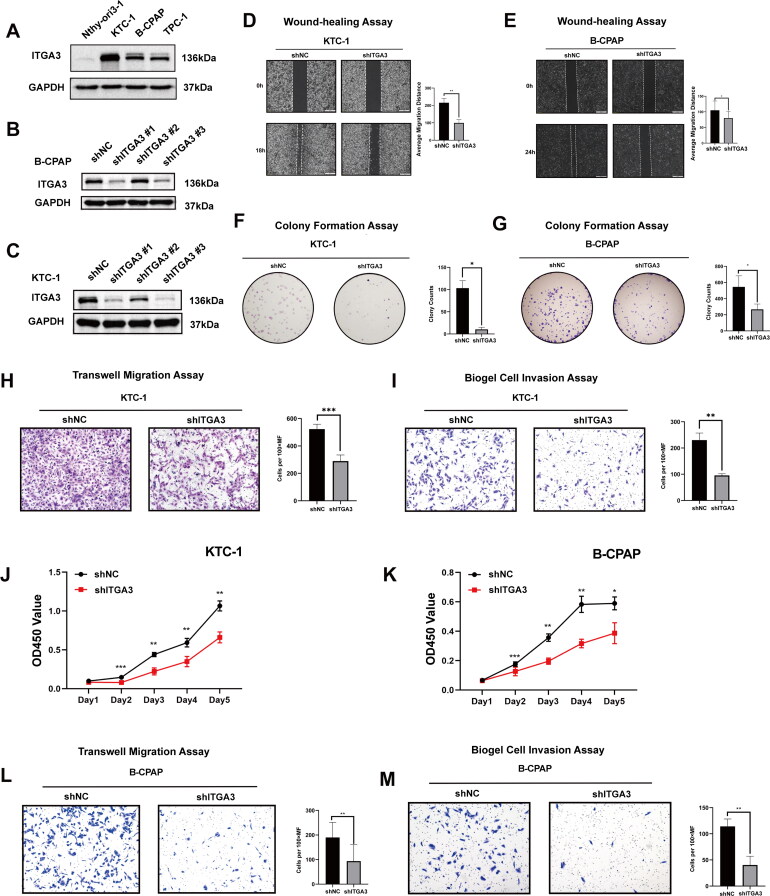
Knockdown of ITGA3 inhibits PTC cell proliferation and migration. (A) Western blot showing the protein level of ITGA3 in nthy-ori3-1 and three other PTC cell lines. (B)-(C) Western blot showing the protein level of ITGA3 in B-CPAP and KTC-1 cells after infection with shNC (scramble shRNA) or shITGA3 lentivirus. (D)-(E) Wound-healing assay showing cell migration was inhibited after knockdown of ITGA3 in KTC-1 and B-CPAP cells. (F)-(G) Colony formation assay showing cell proliferation was inhibited after knockdown of ITGA3 in KTC-1 and B-CPAP cells. (H)-(I) Transwell assay showing cell migration and invasion were inhibited after knockdown of ITGA3 in KTC-1 cells. (J)-(K) CCK8 assay showing cell proliferation was inhibited after knockdown of ITGA3 in KTC-1 and B-CPAP cells. (L)-(M) Transwell assay showing cell migration and invasion were inhibited after knockdown of ITGA3 in B-CPAP cells. (*p < 0.05, **p < 0.01, ***p < 0.001).

**Figure 4.**
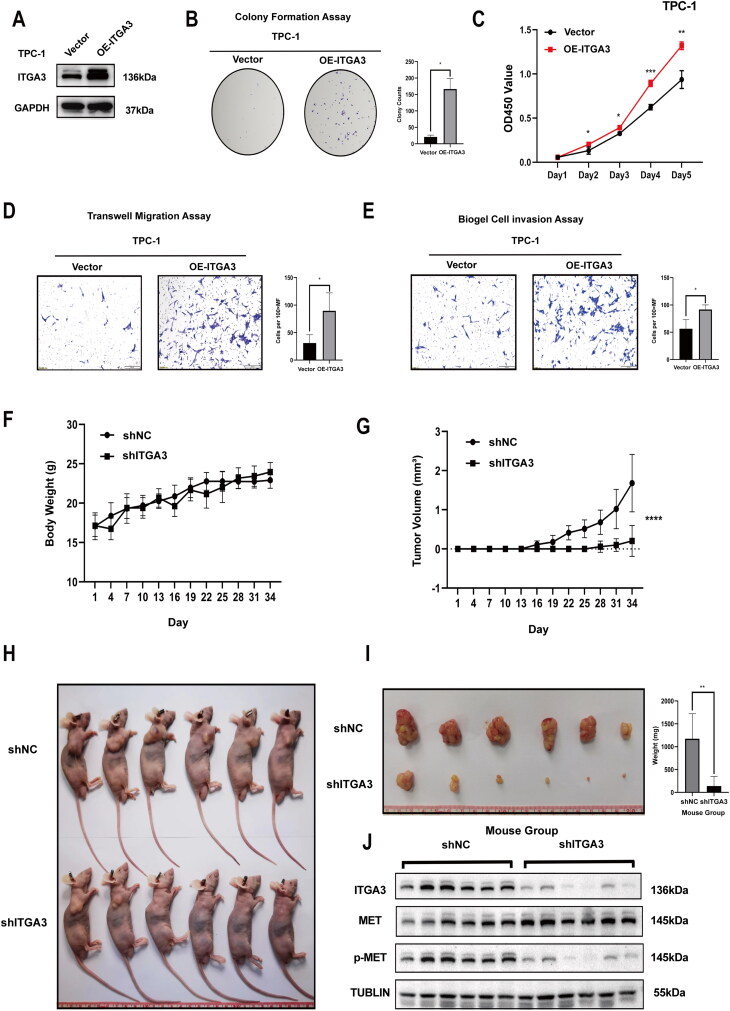
ITGA3 Promotes PTC cell proliferation and migration *in vitro* and *in vivo*. (A) Western blot showing the level of ITGA3 overexpression in TPC-1 cells. (B) Colony formation assay showing cell proliferation was enhanced after overexpression of ITGA3 in TPC-1 cells. (C) CCK8 assay showing cell proliferation was elevated after overexpression of ITGA3 in TPC-1 cells. (D) Transwell assay showing cell migration was enhanced after overexpression of ITGA3 in TPC-1 cells. (E) Transwell assay showing cell invasion was elevated after overexpression of ITGA3 in TPC-1 cells. (F) Body weight curves of mice at different time points. (G) Growth curves of xenograft tumors. (H) Xenograft tumor growth of shNC (scramble shRNA) and ITGA3-knockdown cells. (I) Tumors were removed from nude mice on day 34, and tumor weight was measured. (J) Western blot showing the protein level of ITGA3 in xenograft tumors from shNC (scramble shRNA) and shITGA3 groups. (*p < 0.05, **p < 0.01, ***p < 0.001) mm^3^

Further, we investigated the functional role of ITGA3 in progression and/or tumorigenesis in human PTC. First, relative to Nthy-ori3-1 cells, PTC cells had higher ITGA3 protein expression based on western blotting (Figure 3(A)). Utilizing viral transduction, we established stable cell lines characterized by either ITGA3 knockdown or ITGA3 overexpression. This allowed us to investigate the pivotal role of ITGA3 in tumorigenesis, encompassing both *in vitro* and *in vivo* experimental paradigms (Figure 3(B–C), Figure 4(A)). Knockdown of ITGA3 inhibited migration and proliferation in KTC-1 and B-CPAP cells, as judged by wound-healing assays, cell proliferation assays, transwell migration and invasion assays, and colony formation assays (Figure 3(D–M)). Conversely, overexpression of ITGA3 enhanced the migration and proliferation of TPC-1 cells (Figure 4(B–E)). Additionally, subcutaneous xenograft tumour experiments showed that ITGA3-knockdown cells gave a smaller tumour volume and lower body weight relative to the shNC group (Figure 4(F–J)). These results indicate that ITGA3 promotes PTC proliferation and migration *in vitro* and *in vivo*.

### ITGA3 reduces FAK/src signalling in PTC cells

**Figure 5.**
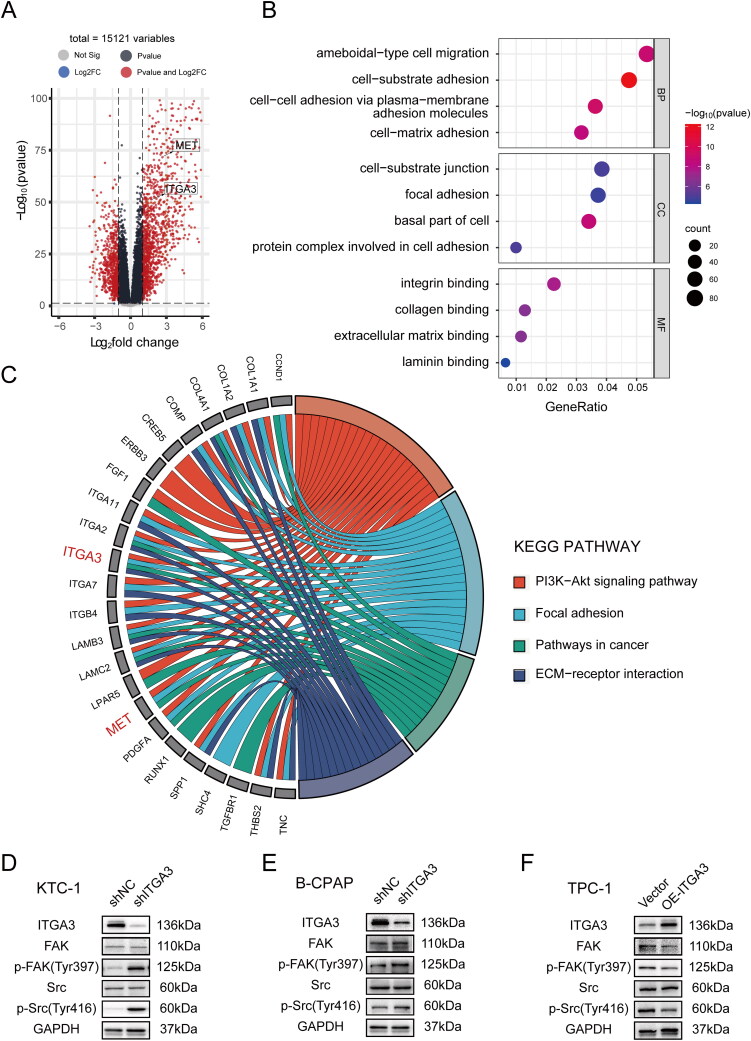
ITGA3 Inhibits the FAK/src pathway in PTC cells. (A) Volcano plots showing differential gene analysis extracted from the THCA cohort in TCGA, with significant genes (red). (B) The top 4 gene ontology (GO) enrichment terms connected to ITGA3 in thyroid cancer clusters, including biological process (BP), cellular component (CC) and molecular function (MF). (C) Kyoto encyclopaedia of genes and genomes (KEGG) pathways connected to ITGA3 in papillary thyroid cancer clusters. (D)-(F) Western blotting showing the protein level of FAK/src signaling after knockdown or overexpression of ITGA3 in PTC cells.

To further characterize the biological function and potential mechanisms accounting for ITGA3-mediated PTC progression, GO and KEGG enrichment analyses were performed. The top four enriched biological processes (BP) that were connected to ITGA3 in the papillary thyroid cancer cluster included ameboidal-type cell migration, cell-substrate adhesion, cell-cell adhesion *via* plasma-membrane adhesion molecules and cell-matrix adhesion, while the cellular component (CC) enrichment included cell-substrate junction, focal adhesion, basal part of cell, protein complex involved in cell adhesion, and molecular function (MF) involved in integrin binding, collagen binding, extracellular matrix binding and laminin binding **(**all *p* < 0.05, Figure 5(A–B)). Furthermore, KEGG pathway analysis identified the PI3K-AKT signalling (*p* = 0.002) and focal adhesion (*p* = 0.003) pathways in cancer (*p* = 0.001), and ECM-receptor interaction (*p* = 7.95E-8) to be connected with ITGA3 (Figure 5(C)). As previous studies have shown that ITGA3 activates FAK and Src signalling, which are involved in cell adhesion, migration, invasion, and proliferation in skin carcinoma [33], bladder cancer [34] and cervical carcinoma [35], we investigated the potential regulation of FAK/Src signalling by ITGA3 in PTC cells. Unexpectedly, our results showed that knockdown of ITGA3 enhanced FAK-Tyr397 and SRC-Tyr416 phosphorylation in KTC-1 and B-CPAP cells, whereas overexpression of ITGA3 in TPC-1 reduced FAK-Tyr397 and SRC-Tyr416 phosphorylation (Figure 5(D–F)). These results suggest that ITGA3 reduces FAK/Src phosphorylation in PTC cells, indicating that the effects of ITGA3 on proliferation and migration of PTC cells may not occur *via* FAK/Src signalling.

### ITGA3 cooperates with MET to regulate downstream signalling pathways via phosphorylation of MET at tyrosine 1234/1235

**Figure 6.**
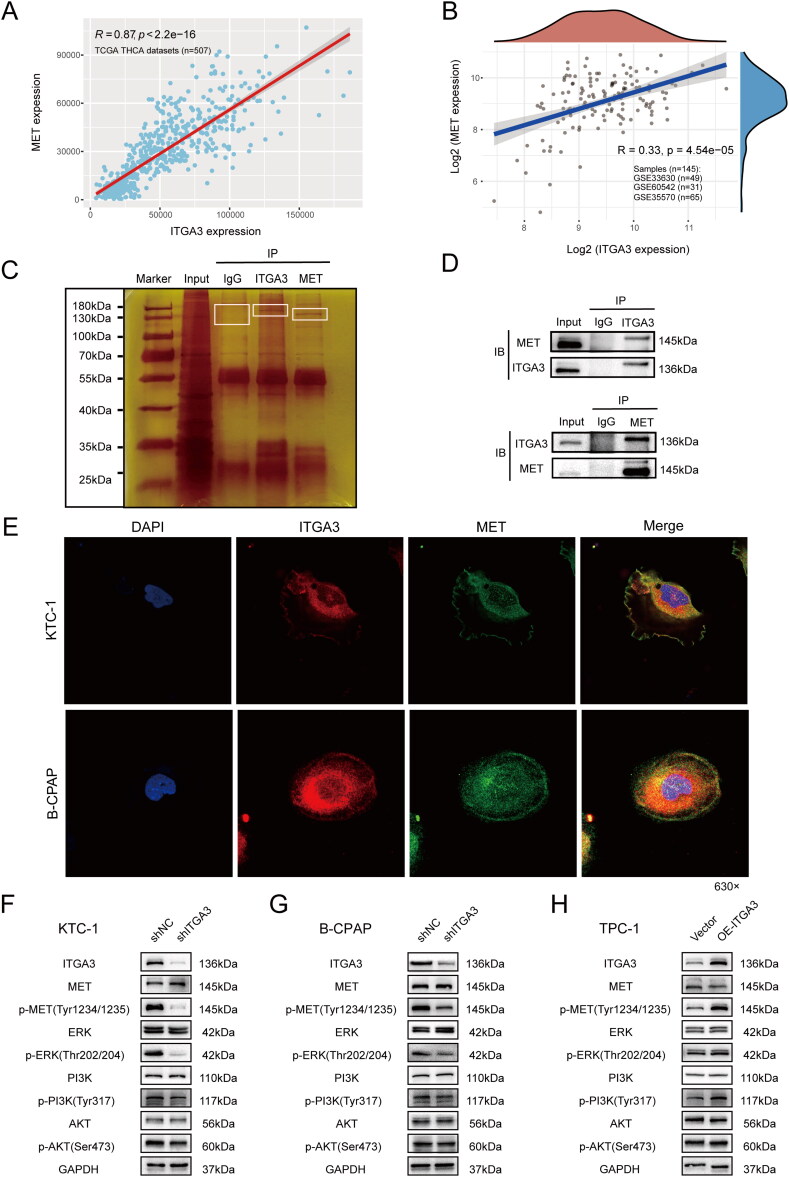
ITGA3 Cooperates with MET to regulate downstream signalling pathways. (A) Scatter plot of the correlation of ITGA3 and MET expression in the THCA cohort from TCGA (*n* = 507). (B) Scatter plot of the correlation of ITGA3 and MET expression in the THCA cohort from the GEO datasets (GSE60542, GSE33630, GSE35570, *n* = 145). (C) Silver staining of immunoprecipitated ITGA3 and MET from KTC-1 cells. (D) Co-immunoprecipitation of ITGA3 and MET in KTC-1 cells. (E) Immunofluorescence of ITGA3 and MET in KTC-1 and B-CPAP cells. (F)-(H) Western blotting showing the protein level of downstream signaling molecules after knockdown or overexpression of ITGA3 in PTC cells.

Further GSEA analysis showed that MET ranked first in the key gene lists of several metastasis-related KEGG pathways (Figure S3A-F). Additionally, bioinformatics analysis indicated that either ITGA3 or MET was upregulated in PTC tissues (Figure 5(A)), and there was good positive correlation between them (*r* = 0.87, *p* < 2.2e-16 in TCGA cohorts, and *r* = 0.33, *p* = 4.54e-15 in GEO datasets) (Figure 6(A–B)). Thus, MET could be one of the key downstream proteins of ITGA3 in PTC. We considered that ITGA3 and MET associate to regulate downstream signalling pathways, given previous reports that integrins and RTKs can interact [20]. Silver staining indicated that proteins in the 130–180 kDa range had ITGA3- or MET-binding candidates by comparing the anti-ITGA3 or anti-MET with anti-IgG IP products of KTC-1 cells (Figure 6(C)). Co-immunoprecipitation assays were then performed to validate the interaction between ITGA3 and MET in total proteins of KTC-1 cells (Figure 6(D)). Moreover, confocal microscopy confirmed the interaction, showing that ITGA3 and MET co-localize on the cell membranes of both KTC-1 or B-CPAP cells (Figure 6(E)). We found that knockdown of ITGA3 resulted in decreased phosphorylation of MET in the activation loop of the tyrosine kinase domain (Tyr1234/1235), but total MET expression was unchanged in KTC-1 and B-CPAP cells (Figure 6(F–G)). Additionally, overexpression of ITGA3 notably increased MET-Tyr1234/1235 phosphorylation without a change in total MET expression in TPC-1 cells (Figure 6(H)). Moreover, we examined whether coordinate signalling by ITGA3 and MET regulates the activation of PI3K/AKT signalling and ERK signalling, as MET and ITGA3 were both enriched in the same KEGG pathways in the thyroid cancer cluster (Figure 5(C)). We observed significant reduction in the phosphorylation of PI3K (Tyr317), AKT (Ser473) and ERK (Thr202/204) upon knockdown of ITGA3 in KTC-1 and B-CPAP cells, whereas overexpression of ITGA3 in TPC-1 cells markedly increased phosphorylation levels of PI3K (Tyr317), AKT (Ser473) and ERK (Thr202/204) (Figure 6(F–H)). Together, these results suggest that signalling through the MET/PI3K/AKT or MET/ERK MAPK pathway is dependent on an interaction with ITGA3 in PTC.

### ITGA3 as an indicator of PTC diagnostic prediction

**Figure 7.**
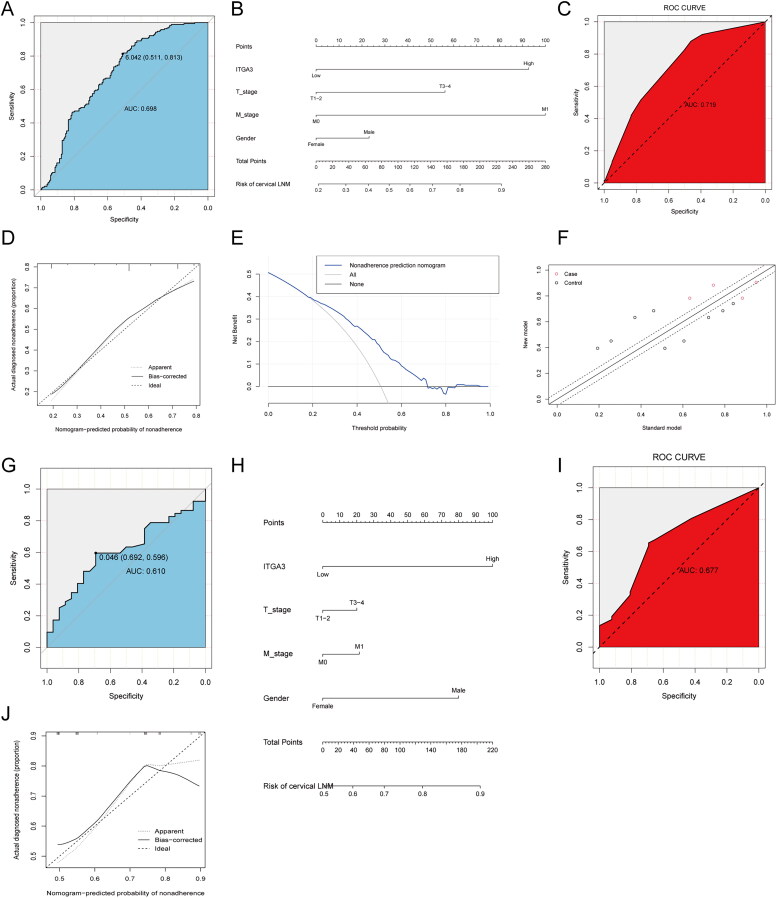
Establishment and validation of a nomogram for predicting PTC cervical lymph node metastasis. (A) Optimal cutoff point of ITGA3 for diagnosing CLNM in the cohort in TCGA. (B) Nomogram for predicting PTC CLNM established by the cohort in TCGA. (C) ROC curve for internal validation. (D) Calibration curve for internal validation. (E) DCA curve for internal validation. (F) Net reclassification index for internal validation. (G) Optimal cutoff point of ITGA3 for diagnosing CLNM in the SUMC cohort. (H) Nomogram for predicting CLNM established by the SUMC cohort. (I) ROC curve for external validation. (J) Calibration curve for external validation.

Since ITGA3 was closely correlated with cell migration in PTC, we established a diagnostic prediction model for predicting CLNM. First, PTC patients from TCGA were divided into high- and low-ITGA3 groups by the optimal cutoff point of ITGA3 (Figure 7(A)). Gender, ITGA3 level, T stage and M stage were entered into multivariate logistic regression to establish a diagnostic prediction model (Table 4). Then, a nomogram was constructed to facilitate clinical use of the model (Figure 7(B)). In the current study, the C-index, AUC and NRI were 0.719, 0.719 and 0.623, respectively, indicating good prediction accuracy of the fitted model (Figure 7(C,E)). The calibration plot-predicted CLNM of PTC patients also performed well when compared with the ideal model (Figure 7(D)), and the DCA curve indicated good diagnostic value of the current diagnostic model (Figure 7(F)). Additionally, the external validation set yielded an AUC of 0.677, which implied good predictive accuracy of the current prediction model (Figure 7(G–J)).

**Table 4.** Univariate and multivariate logistic analysis of risk factors for CLNM in PTC patients.

Parameters	Univariate logistic			Multivariate logistic		
	OR^a^	CI^b^	*P*	OR	CI	*P*
**Age**	0.92	0.59–1.42	0.69	NA	NA	NA
**Focus Type**	1.13	0.75–1.69	0.56	NA	NA	NA
**Gender**	0.66	0.42–1.06	0.09	0.69	0.41–1.16	0.1584
**ITGA3**	4.4	2.77–6.99	<0.001	4.42	2.73–7.15	<0.001
**M Stage**	4.97	0.58–42.98	0.14	4.96	0.51–48.23	0.1671
**T Stage**	2.73	1.78–4.2	<0.001	2.46	1.56–3.89	0.0001

^a^OR: Odds ratio. ^b^CI: Confidence interval.

## Discussion

Papillary thyroid carcinoma is the most common type of thyroid cancer. Jain et al. [10] and Liu et al. [11] showed that higher integrin α3 expression plays a key role in PTC initiation and progression. Mautone et al. [12] demonstrated integrin α3 expression displays the highest correlation with advanced disease, and integrin α3β1 is associated with negative prognosis in PTC. However, the altered ITGA3 signalling pathways in PTC have not been explored.

As integrins do not belong to the kinase family, integrin signalling activation requires the recruitment of cytoplasmic kinases, such as FAK and ILK. FAK is a typical example of a protein that is highly phosphorylated in response to integrin activation and has diverse cellular functions, including cell proliferation, adhesion and migration, that impinge on tumour cell behaviour [33–36]. Integrin α3β1 has been shown to initiate FAK signalling and promote malignant proliferation and invasion in cancers such as breast cancer [37], ovarian cancer [22], head and neck cancer [23,24] and pancreatic cancer [18,25,38]. Our study shows that upregulation of integrin α3 reduces FAK-Tyr397 and Src-Tyr416 phosphorylation in PTC, which is different from what has been reported above.

Previous studies have reported the crosstalk between integrins and MET [20,26–28]. MET-integrin cooperation can occur through outside-in signalling, whereby integrin activation, triggered by integrin binding to its extracellular ligand in the ECM, thus promoting cell-surface adhesion, is also able to induce MET phosphorylation [20,26]. The integrin acts upstream of MET in an HGF-independent manner such that treating or plating ovarian, breast, lung or prostate cancer cells with or on integrin substrates, such as fibronectin, collagen or laminin, triggers MET phosphorylation in various cell models [20,26,27,39–41]. The exact mechanisms of MET phosphorylation downstream of an integrin, however, are not clear.

Integrin α3 has also been reported to exert its effects through the MAPK signalling pathway [42] and interaction with other molecules, such as tetraspanins [43]. In our study, we demonstrate that integrin α3 upregulates MET activity, leading to subsequent activation of the ERK1/2 and PI3K/AKT signalling pathways. This results in enhanced progression, migration, and invasion of PTC cells. These findings suggest that the cooperation between MET and ITGA3 could serve as a promising target for combination therapy, particularly in cases of drug resistance to monotherapy.

Surgical resection with prophylactic central lymph node dissection (CLND) is considered effective for treating PTC [44–47]. However, due to potential risks, such as hypoparathyroidism and nerve injury [44,45,48], some clinicians prefer therapeutic CLND. Therefore, accurately assessing CLNM risk is crucial in deciding whether prophylactic CLND can be performed. We developed a nomogram that combines ITGA3 expression and clinical parameters (gender, T stage, and M stage) to predict CLNM in PTC patients. The nomogram showed excellent prediction accuracy in both the fitted model and the external validation set from our cohort. These results suggest that ITGA3 might serve as a diagnostic predictor of PTC.

To summarize, we have uncovered a potential mechanism in which the interaction between overexpressed ITGA3 and MET facilitates the progression of PTC through the ERK and PI3K/AKT pathways. However, we have noted a limitation of this study is that the mechanism underlying ITGA3 overexpression in PTC remains to be further explored.

## Conclusions

In PTC, upregulated ITGA3 reduces FAK/Src phosphorylation, and also interacts directly with MET to promote MET phosphorylation in an HGF-independent manner, followed by activation of the ERK1/2 and PI3K/AKT signaling pathways, and promotion of PTC cell progression, migration and invasion. ITGA3 could serve as an indicator of PTC diagnostic prediction, and MET-ITGA3 cooperation may act as a potential target for combination therapy.

## Supplementary Material

Supplemental Material

## Data Availability

The Cancer Genome Atlas (TCGA) thyroid cancer dataset (THCA) used in this study can be obtained from TCGA (https://portal.gdc.cancer.gov/). The Gene Expression Omnibus (GEO) database (GSE60542, GSE33630, GSE35570) were downloaded from the GEO database (https://www.ncbi.nlm.nih.gov/gds). This article was based on a secondary analysis of published literature and data from public databases, and no new data had been generated. The raw data files supporting the findings from our own experiments in this study were available from the corresponding author, Muyuan Liu, upon reasonable request.
