# Supplementary material for: ITGA3–MET interaction promotes papillary thyroid cancer progression via ERK and PI3K/AKT pathways
Source: Ann Med. 2025 Mar 26;57(1):2483379. doi: 10.1080/07853890.2025.2483379 (PMC11948363; doi:10.1080/07853890.2025.2483379)
Supplement: Supplemental Material [file IANN_A_2483379_SM4527.zip › suppl_data/Suppl_Fig caption.docx]

**Figure S1.** Dependence of disease-free survival on hub genes. (A) ITGA3 disease-free survival. (B) RUNX1 disease-free survival. (C) PTPRE disease-free survival. (D) MET disease-free survival. (E) KCNQ3 disease-free survival. (F) TNFRSF10A disease-free survival. (G) MBOAT2 disease-free survival. (H) SHROOM4 disease-free survival. (I) CDC42BPG disease-free survival. (J) IL1RAP disease-free survival.

**Figure S2.** ITGA3 is upregulated in multiple cancers.

**Gene set enrichment analysis**

To explore ITGA3-related signaling pathways, single-gene gene set enrichment analysis (GSEA) was conducted. First, thyroid cancer patients were classified into high- and low-ITGA3 groups by the median mRNA level of ITGA3 in the TCGA THCA dataset. GSEA version 4.0.2 was used to identify the significantly enriched gene sets. *P* < 0.01 and a false discovery rate (FDR) <25% were considered statistically significant. The GSEA results showed 131 gene sets were significantly enriched in the ITGA3 high-expression cluster. The top 30 enriched gene sets are shown in Table S3, and most of them were strongly correlated with metastasis, including endocytosis (FDR = 0.001), adherens junction (FDR = 0.003), pathway in cancer (FDR = 0.004), epithelial cell signaling in helicobacter pylori infection (FDR = 0.006), cytokine receptor interaction (FDR = 0.006) and focal adhesion (FDR = 0.006). Among them, the MET oncogene ranked first in the key gene lists of these four pathways (Figure S3).

**Figure S3. GSEA analysis of KEGG pathways based on ITGA3 expression in thyroid cancer patients.** (A) Endocytosis. (B) Adherens junction. (C) Pathway in cancer. (D) Epithelial cell signaling in helicobacter pylori infection. (E) Cytokine receptor interaction. (F) Focal adhesion.
