# Supplementary material for: ITGA3–MET interaction promotes papillary thyroid cancer progression via ERK and PI3K/AKT pathways
Source: Ann Med. 2025 Mar 26;57(1):2483379. doi: 10.1080/07853890.2025.2483379 (PMC11948363; doi:10.1080/07853890.2025.2483379)
Supplement: Supplemental Material [file IANN_A_2483379_SM4527.zip › suppl_data/Table S1.docx]

**Table S1. Primary antibodies for western blotting (WB) and co-immunoprecipitation (Co-IP).**

| **Antibody** | **Dilution** | **Catalog number** | **Company** |
| --- | --- | --- | --- |
| ITGA3 | 1:1000 (WB) 3ug (IP) | PA5-82356 | Invitrogen |
| FAK | 1:1000 (WB) | 66258-1-Ig | Proteintech |
| p-FAK(Tyr397) | 1:1000 (WB) | 8556 | Cell Signaling Technology |
| SRC | 1:300 (WB) | 11097-1-AP | Proteintech |
| p-SRC(Tyr416) | 1:1000 (WB) | 6943 | Cell Signaling Technology |
| MET | 1:1000 (WB)  3ug (IP) | 71-8000 | Thermo Fisher |
| p-Met (Tyr1234/1235) | 1:1000 (WB) | 3077 | Cell Signaling Technology |
| p44/42 MAPK (Erk1/2) | 1:1000 (WB) | 9102S | Cell Signaling Technology |
| p-p44/42 MAPK (Erk1/2) | 1:1000 (WB) | 4370S | Cell Signaling Technology |
| GAPDH | 1:1000 (WB) | sc-137179 | Santa Cruz |
| β-actin | 1:2000 (WB) | Sc-130656 | Santa Cruz |
| α-tubulin | 1:10000 (WB) | ab7291 | Abcam |
| Normal Rabbit IgG | 1ug (IP) | 2729 | Cell Signaling Technology |
| PI3K | 1:1000 (WB) | AF5112 | affinity |
| p-PI3K (Tyr317) | 1:2000 (WB) | bs5570r | Bioss |
| AKT | 1:10000 (WB) | ab179463 | Abcam |
| p-AKT (Ser473) | 1:1000 (WB) | AF0016 | affinity |
