# Supplementary material for: ITGA3–MET interaction promotes papillary thyroid cancer progression via ERK and PI3K/AKT pathways
Source: Ann Med. 2025 Mar 26;57(1):2483379. doi: 10.1080/07853890.2025.2483379 (PMC11948363; doi:10.1080/07853890.2025.2483379)
Supplement: Supplemental Material [file IANN_A_2483379_SM4527.zip › suppl_data/Table S2.docx]

**Table S2. List of shRNA sequences.**

| **shRNA** | **sequence** |
| --- | --- |
| shITGA3#1 | 5′-GCAATAGCAACACAGACTACC-3′ |
| shITGA3#2 | 5′-GCAGTGAGTCCGCTGTCTTCC-3′ |
| shITGA3#3 | 5′-GCAGAGACGTCCGGAAATTGC-3′ |
| shNC (scramble shRNA) | 5′-TTCTCCGAACGTGTCACGT-3′ |
