# Supplementary material for: ITGA3–MET interaction promotes papillary thyroid cancer progression via ERK and PI3K/AKT pathways
Source: Ann Med. 2025 Mar 26;57(1):2483379. doi: 10.1080/07853890.2025.2483379 (PMC11948363; doi:10.1080/07853890.2025.2483379)
Supplement: Supplemental Material [file IANN_A_2483379_SM4527.zip › suppl_data/Table S3.docx]

**Table S3. Top 30 enriched gene sets in the high-ITGA3 group.**

| **NAME** | **SIZE** | **ES** | **NES** | **NOM p-val** | **FDR q-val** |
| --- | --- | --- | --- | --- | --- |
| KEGG_NATURAL_KILLER_CELL_MEDIATED _CYTOTOXICITY | 132 | 0.682 | 2.099 | 0.00 | 0.000 |
| KEGG_ENDOCYTOSIS | 179 | 0.692 | 2.072 | 0.00 | 0.001 |
| KEGG_REGULATION_OF_ACTIN_CYTOSKELETON | 212 | 0.643 | 2.062 | 0.00 | 0.001 |
| KEGG_P53_SIGNALING_PATHWAY | 67 | 0.720 | 2.042 | 0.00 | 0.001 |
| KEGG_VIRAL_MYOCARDITIS | 68 | 0.750 | 2.031 | 0.00 | 0.002 |
| KEGG_AXON_GUIDANCE | 128 | 0.651 | 2.025 | 0.00 | 0.002 |
| KEGG_TIGHT_JUNCTION | 129 | 0.652 | 2.019 | 0.00 | 0.001 |
| KEGG_PATHOGENIC_ESCHERICHIA_COLI_INFECTION | 56 | 0.742 | 2.019 | 0.00 | 0.001 |
| KEGG_CELL_ADHESION_MOLECULES_CAMS | 128 | 0.664 | 1.997 | 0.00 | 0.002 |
| KEGG_LEUKOCYTE_TRANSENDOTHELIAL_MIGRATION | 115 | 0.642 | 1.994 | 0.00 | 0.002 |
| KEGG_JAK_STAT_SIGNALING_PATHWAY | 155 | 0.598 | 1.986 | 0.00 | 0.001 |
| KEGG_APOPTOSIS | 87 | 0.680 | 1.962 | 0.00 | 0.002 |
| KEGG_CHEMOKINE_SIGNALING_PATHWAY | 186 | 0.628 | 1.962 | 0.00 | 0.002 |
| KEGG_LYSOSOME | 121 | 0.702 | 1.957 | 0.00 | 0.002 |
| KEGG_CELL_CYCLE | 124 | 0.707 | 1.948 | 0.00 | 0.002 |
| KEGG_CHRONIC_MYELOID_LEUKEMIA | 73 | 0.706 | 1.937 | 0.00 | 0.003 |
| KEGG_ADHERENS_JUNCTION | 68 | 0.719 | 1.929 | 0.00 | 0.003 |
| KEGG_PATHWAYS_IN_CANCER | 322 | 0.587 | 1.918 | 0.00 | 0.004 |
| KEGG_RIG_I_LIKE_RECEPTOR_SIGNALING_PATHWAY | 70 | 0.626 | 1.914 | 0.00 | 0.004 |
| KEGG_ARRHYTHMOGENIC_RIGHT_VENTRICULAR_CARDIOMYOPATHY_ARVC | 74 | 0.615 | 1.906 | 0.00 | 0.004 |
| KEGG_DNA_REPLICATION | 36 | 0.820 | 1.903 | 0.00 | 0.004 |
| KEGG_TOLL_LIKE_RECEPTOR_SIGNALING_PATHWAY | 102 | 0.616 | 1.882 | 0.01 | 0.006 |
| KEGG_CYTOSOLIC_DNA_SENSING_PATHWAY | 54 | 0.603 | 1.879 | 0.00 | 0.006 |
| KEGG_EPITHELIAL_CELL_SIGNALING_IN  _HELICOBACTER_PYLORI_INFECTION | 68 | 0.652 | 1.878 | 0.00 | 0.006 |
| KEGG_CYTOKINE_CYTOKINE_RECEPTOR_INTERACTION | 263 | 0.575 | 1.872 | 0.01 | 0.006 |
| KEGG_FOCAL_ADHESION | 197 | 0.603 | 1.869 | 0.00 | 0.006 |
| KEGG_LEISHMANIA_INFECTION | 70 | 0.711 | 1.865 | 0.00 | 0.006 |
| KEGG_NOD_LIKE_RECEPTOR_SIGNALING_PATHWAY | 61 | 0.650 | 1.864 | 0.00 | 0.006 |
| KEGG_FC_GAMMA_R_MEDIATED_PHAGOCYTOSIS | 96 | 0.621 | 1.851 | 0.00 | 0.007 |
| KEGG_HOMOLOGOUS_RECOMBINATION | 26 | 0.764 | 1.844 | 0.00 | 0.007 |
