# Supplementary figures and images for: ITGA3–MET interaction promotes papillary thyroid cancer progression via ERK and PI3K/AKT pathways
Source: Ann Med. 2025 Mar 26;57(1):2483379. doi: 10.1080/07853890.2025.2483379 (PMC11948363; doi:10.1080/07853890.2025.2483379)

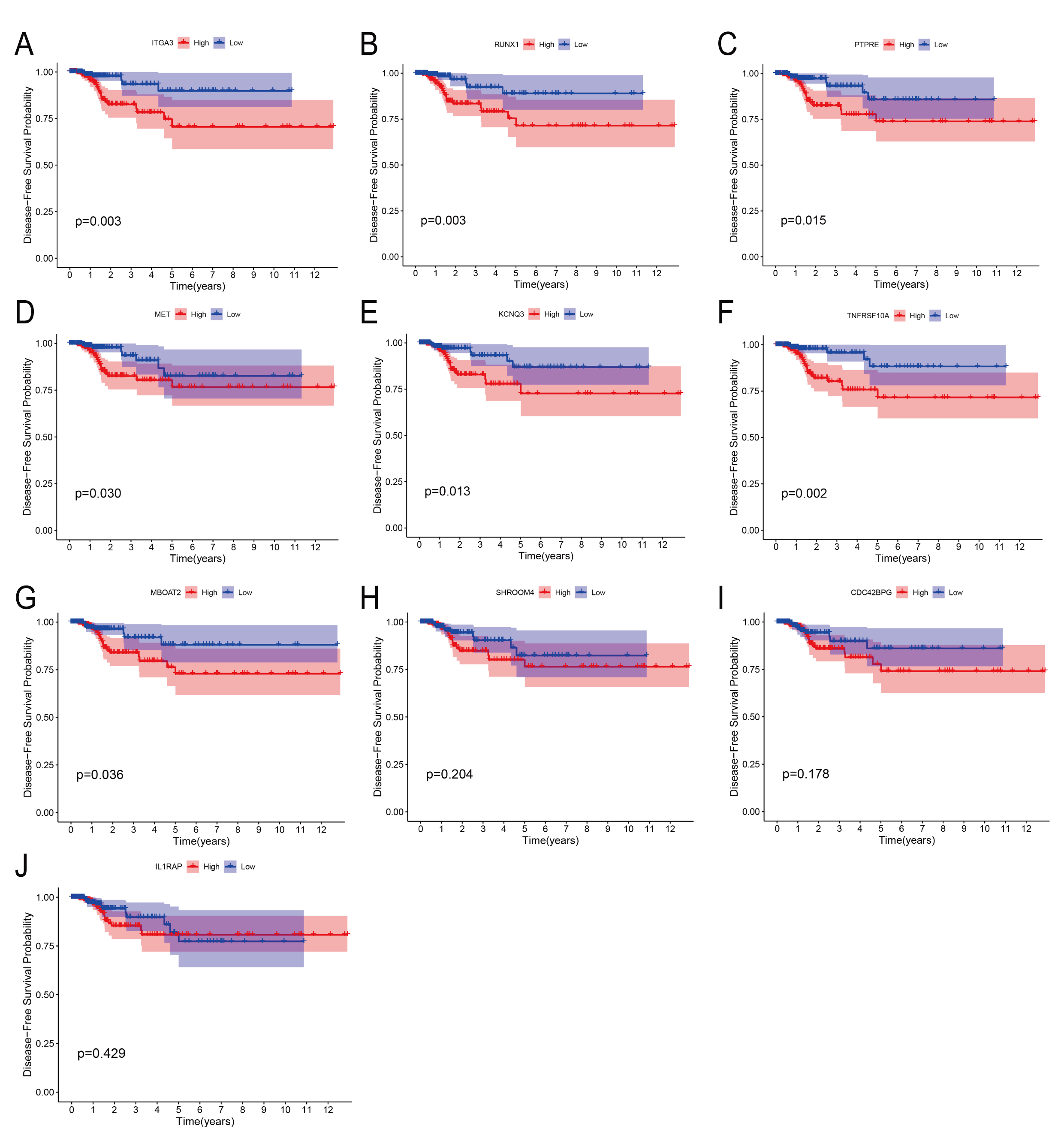

Supplement: Supplemental Material [file IANN_A_2483379_SM4527.zip › suppl_data/Figure S1.tif]

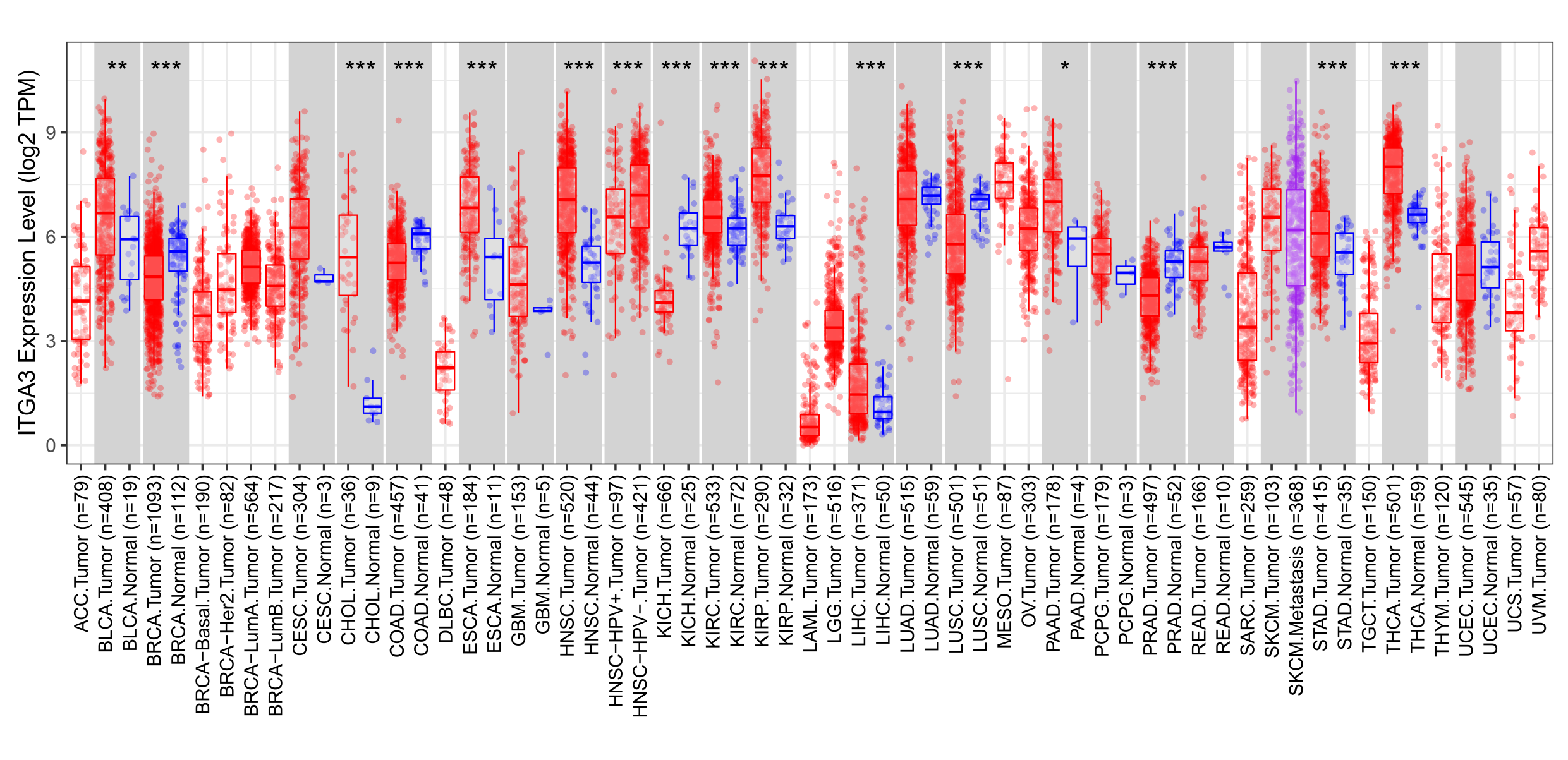

Supplement: Supplemental Material [file IANN_A_2483379_SM4527.zip › suppl_data/Figure S2.tif]

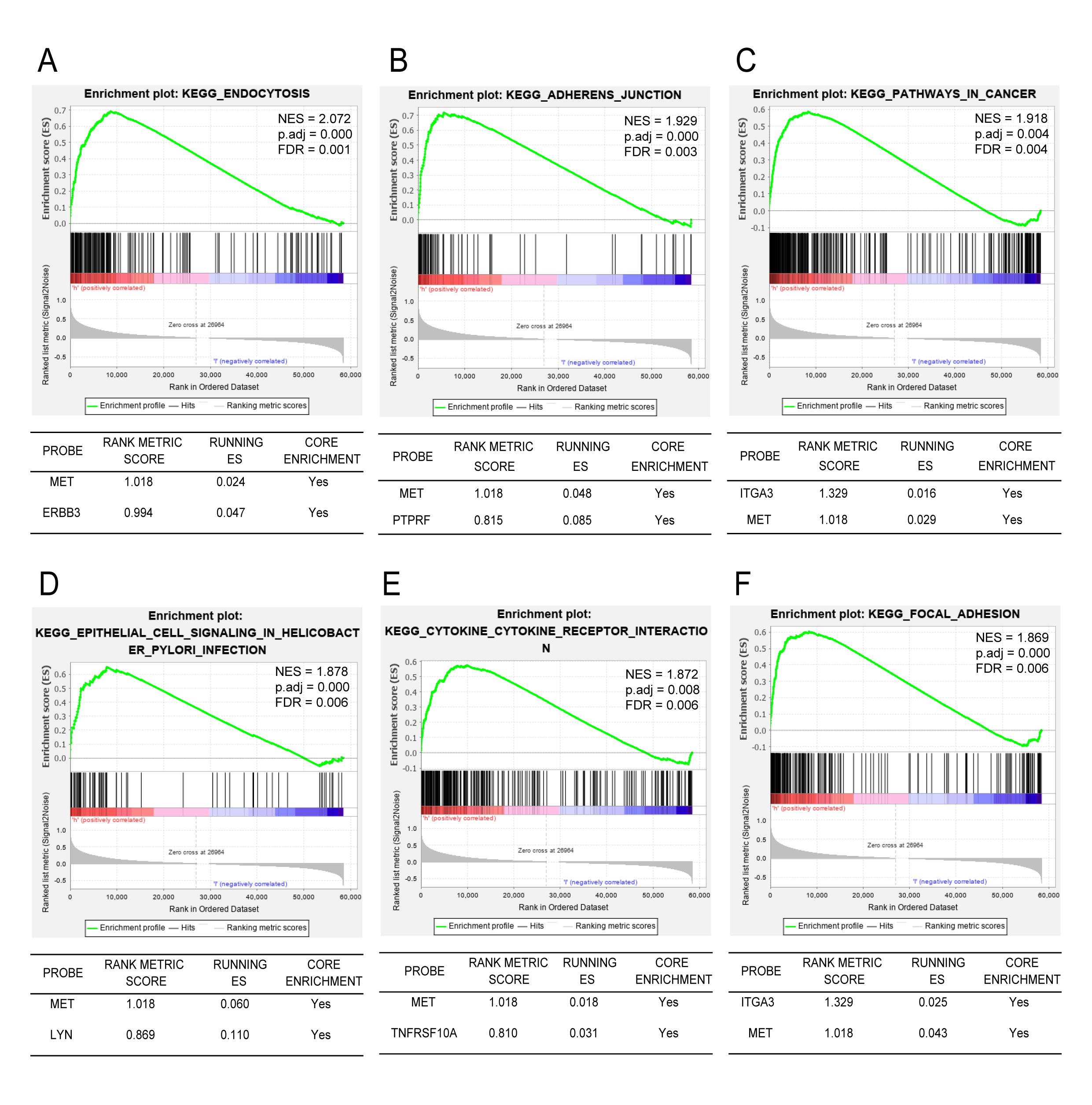

Supplement: Supplemental Material [file IANN_A_2483379_SM4527.zip › suppl_data/Figure S3.tif]
